# Leech Extracts and Thrombosis

**Published:** 1895-04-06

**Authors:** 


### THERAPEUTIC NOTES.

### LEECH EXTRACT AND THROMBOSIS.

Some years ago Haycraft showed that leeches secrete a substance which, when injected into the blood of other animals, hinders or prevents coagulation, and that, owing to this, leech bites are very apt to bleed profusely. Sahli has investigated the effect of leech extract on the formation of thrombi (Cbl. f. inn. Med., June, 1894), a bristle being passed into the external jugular vein to coagulate the blood locally. Normally a thrombus forms on the bristle in about ten minutes, but after varying quantities of a watery extract of leech had been previously injected, thrombosis did not occur. The duration of the preventive effect is limited to hours. The infusion of leech is not poisonous, and one leech suffices for each two ounces of blood, but large quantities have not been tried. Not less than eighty or ninety leeches would be required for a man. As yet no practical therapeutical results have been obtained.

